# Immediate effect of passive hamstring stretching on flexibility and relationship with psychosocial factors in people with chronic low back pain

**DOI:** 10.1016/j.heliyon.2023.e19753

**Published:** 2023-09-04

**Authors:** Lech Dobija, Bruno Pereira, Gabriel Cohen-Aknine, Alexandra Roren, Arnaud Dupeyron, Emmanuel Coudeyre

**Affiliations:** a Service de Médecine Physique et de Réadaptation, Centre Hospitalier Universitaire (CHU) de Clermont Ferrand, 63000 Clermont-Ferrand, France; b Unité de Nutrition Humaine, INRAE, Université Clermont Auvergne, 63000 Clermont-Ferrand, France; c Direction de la Recherche Clinique et de l’Innovation, Centre Hospitalier Universitaire (CHU) de Clermont-Ferrand, 63000 Clermont-Ferrand, France; d Service de Médecine Physique et de Réadaptation, Centre Hospitalier Universitaire (CHU) de Nîmes, 30900 Nîmes, France; e Service de Rééducation et de Réadaptation de l'Appareil Locomoteur et des Pathologies du Rachis, Assistance Publique-Hôpitaux de Paris (AP-HP) Centre-Université de Paris, Hôpital Cochin, Paris, France

**Keywords:** Chronic low back pain, Hamstring muscles, Muscle stretching, Psychosocial factors, Active knee extension, Straight leg raise

## Abstract

**Background:**

Hamstring muscle tightness contributes to disability in people with chronic low back pain (CLBP). HM stretching improves flexibility in healthy individuals, but the immediate effect of stretching is unknown in people with CLBP. Moreover, the stretching effect could be influenced by psychosocial factors.

**Objectives:**

To evaluate the immediate effect of passive HM stretching on flexibility in people with CLBP and the relationships between psychosocial factors and change in hamstring flexibility.

**Design:**

Non-randomized, pilot trial.

**Method:**

One minute of passive stretching was performed in 90 people with CLBP. Change in Active Knee Extension and Straight Leg Raise angles (digital inclinometer), and Fingertips-to-Floor distance (measuring tape) were measured before and immediately after stretching. Correlations between change in flexibility and baseline Fear-Avoidance Beliefs Questionnaire (FABQ) and Hospital Anxiety and Depression Scale (HADS) scores were analyzed.

**Results:**

Hamstring flexibility improved significantly after stretching; Active Knee Extension mean difference was 4° (95% CI, 2.4 to 5.1; p < 0.001, right ES = 0.24, left ES = 0.23); Straight Leg Raise mean difference was 7° (95% CI, 5.5 to 8.6, p < 0.001, right ES = 0.44, left ES = 0.42), Fingertips-to-Floor mean difference was 2 cm (95% CI, 1.7 to 3.0, p < 0.001, ES = 0.20). No correlation was found between improvement in any of the hamstring flexibility measurements and FABQ or HADS scores (p > 0.05).

**Conclusions:**

Passive hamstring stretching induced an immediate, statistically significantly improvement in hamstring flexibility, but only the change in Straight Leg Raise amplitude was clinically important. Psychosocial factors were not related to improvements in flexibility after hamstring stretching.

Providing optimal treatment for people with chronic low back pain (CLBP) is a major challenge for physical therapists and healthcare professionals. Despite the progress that has been made in physical therapy [[1], [2], [3]], CLBP remains the leading cause of disability [4]. The bio-psycho-social framework addresses the complexity of CLBP by targeting factors that more broadly contribute to the problem [5,6]. Furthermore, the consideration of psychosocial aspects within biomechanical treatment concepts could improve the management of CLBP.

One biomechanical factor that impacts functional capacity is limitation in range of motion (ROM). For example, forward bending during housework or professional activities could be limited by hamstring muscle (HM) tightness. Forward bending involves anterior pelvic tilt and elongation of the HM [7]. However, HM tightness can limit anterior pelvic tilt [8,9] and could contribute to disability in people with CLBP. Improvement in forward bending ROM during the first month after low back pain onset is a valid predictor of functional recovery at one year [10]. Furthermore, the posterior longitudinal muscles connect the HM to the erector spinae muscle via the sacrotuberal ligament and produce compression forces that enhance lumbopelvic stability [[11], [12], [13]]. It has been suggested that reduced HM flexibility could result from poor core muscle function [14,15]. Therefore, HM tightness could be a sub-optimal compensatory mechanism for a lack of core stability that further increases the risk of development of CLBP [16].

Repeated HM stretching sessions improve HM flexibility [17]. Moreover, in healthy people, the result is immediate [18,19]. When combined with core stability exercises, repeated HM stretching improves physical function and decreases pain in people with CLBP [20]. In people with CLBP, pain and fear of pain, fear of movement, and anxiety may impact on the effect of HM stretching. To our knowledge, only one study has evaluated the immediate effect of HM stretching in people with CLBP, showing a positive effect, however only the sit-and-reach test was evaluated, and the sample was small [21].

The immediate effect of a manual therapy intervention provides important information to the physical therapist. It can guide subsequent treatment choices and help to establish a functional diagnosis. For example, if forward bending ROM does not improve after HM stretching, other structures (eg, the piriformis muscle, sciatic nerve or lumbar spine) could be considered as the drivers of the limitation, and could be evaluated, treated and reevaluated to refine the diagnosis. In contrast, if ROM improves immediately, the same technique could be repeated in the following sessions to obtain a cumulative effect. However, if no improvement occurs with repeated interventions, and no red flags are present [1,22], psychosocial or behavioral factors may be involved in the limitation. In people with CLBP, fear-avoidance beliefs and the person's perception that movement is a threat can limit the capacity to relax muscles during stretching, which could limit stretching efficacy [5,23]. Similarly, symptoms of anxiety or depression could reduce the stretching effect [24]. Identifying a relationship between psychosocial factors and the effect of HM stretching could help clinicians to determine the need to address psychosocial factors before performing a stretching intervention. However, no study has evaluated this relationship.

The primary aim of our study was to evaluate the immediate effect of passive HM stretching on HM flexibility in people with CLBP. The secondary aim was to analyze the relationship between psychosocial factors and change in HM flexibility after stretching. We hypothesized that HM flexibility would improve after one session of passive HM stretching, but that improvements would be smaller in people with higher levels of fear-avoidance beliefs, anxiety or depression reported at baseline.

## 1 Methods

### 1.1 Study design

**Fig. 1:**
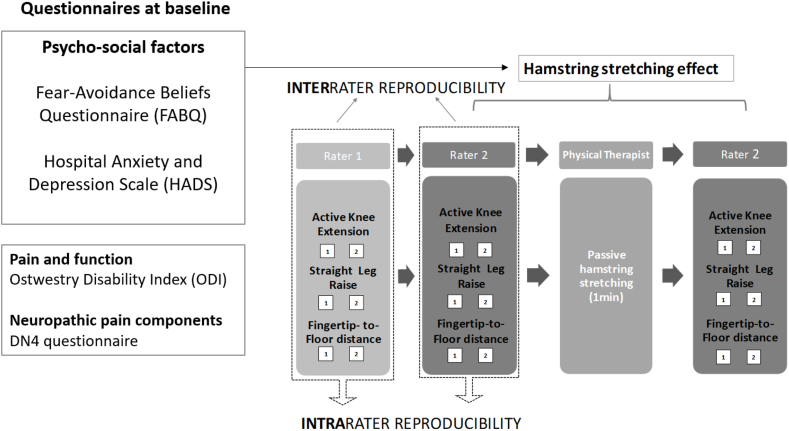
Schema of the study design.

This multicenter, non-randomized, pilot clinical trial was approved by our local ethics committee (Comité de Protection des Personnes – Ouest 1, Identifier: 2020T2-01_RIPH2 HPS_2019-A03000-57). Informed consent was obtained from all participants, including consent for the publication of photographs of them. The study is reported according to the CONSORT guidelines, adapted for our study design (Fig. 1). The study was registered on the ClinicalTrials.gov database (Identifier: NCT04551326). The study schedule is shown in Fig. 1.

### 1.2 Participants

People with CLBP according to the definition in current clinical guidelines [25] and corresponding to the MG30.02 International Classification of Diseases code were recruited from referrals to the physical and rehabilitation medicine departments of two French University Hospitals. Informed consent was obtained from all participants. The inclusion criteria were age between 18 and 60 years, low back pain for more than three months with or without associated pain in the buttock or thigh, reduced HM flexibility (Fingertip-to-Floor [FTF] distance more than 5 cm and Active Knee Extension [AKE] less than 80°). People were not included if they had neurologic, cardiac, respiratory or oncological disease, a history of significant surgery (ex. hip or knee arthroplasty, arthrodesis of more than two vertebral segments), discopathy Modic 1, or radicular pain, were pregnant or breastfeeding women, had restricted mental/legal autonomy or were not covered by the social security system.

### 1.3 Evaluation procedure

**Fig. 2:**
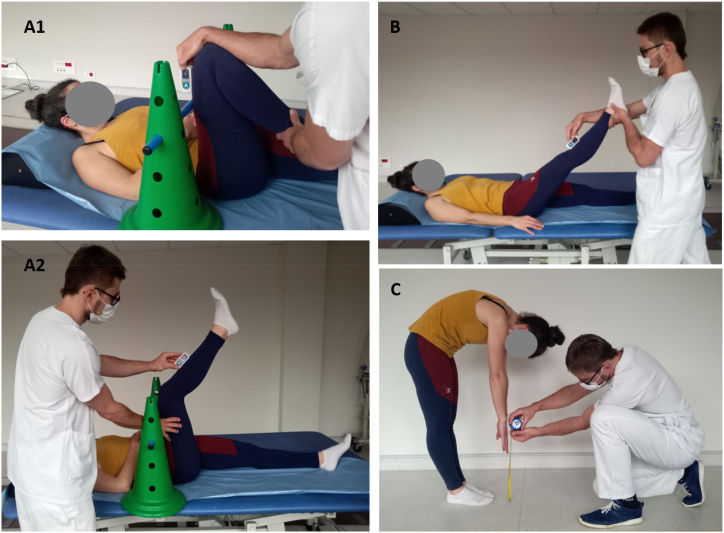
Measurement procedure: (A1, A2) Active Knee Extension angle, (B) Straight Leg Raise angle, (C) Fingertip-to-Floor distance.

All participants were allocated to the same study procedure. Three HM flexibility measurements were performed by two raters: AKE, Straight Leg Raise (SLR) and FTF (Fig. 1). We used the EasyAngle® digital inclinometer for AKE and SLR measurements. Because this is a novel method for these measurements, we performed a preliminary evaluation of the intra- and interrater reliability and construct validity of the AKE and SLR angle measurements in 90 people with CLBP [26]. First, Rater 1 performed the AKE, SLR and FTF twice each. Then, Rater 2 also performed them twice each. Both raters were blinded to the other's measurement. One minute of passive HM stretching was then applied bilaterally by a different physical therapist (see below). After HM stretching, Rater 2 repeated AKE, SLR and FTF measurements twice each. The order of the measurements (AKE, SLR, and FTF) was randomized. Participants were asked to walk for about 30 s when the rater changed and between the stretching and the measurements. The whole study procedure was performed on the same day and the intervention session lasted between 30 and 45 min for each participant. The raters were physical therapists or medical doctors who all had experience in CLBP treatment and were trained in the measurement procedures and the study protocol. The measurements procedures are presented in Fig. 2.

#### 1.3.1 Active Knee Extension

AKE was measured with the participant lying in supine on the examination table. The EasyAngle® digital inclinometer was first calibrated to zero degrees on the surface of the examination table. Then, the participant was instructed to place their thigh vertically. The 90-degree angle was verified with the inclinometer placed on the distal anterior surface of the participant's thigh. In addition, the participant was asked to keep their thigh against a plastic stick placed against the anterior surface of their thigh to ensure that it remained vertical during the measurement. The contralateral limb rested in extension and was stabilized by the rater's hand if necessary. The participant was asked to extend the tested knee as much as possible. The rater placed the inclinometer on the tibial crest, directly below the tibial tuberosity to record the maximal knee extension angle (Fig. 2, A1, A2).

AKE angle is frequently used to evaluate HM flexibility. It has acceptable reliability and agreement in healthy individuals using a universal goniometer [27]. Our reliability assessment in the 90 participants with CLBP found excellent intrarater reliability, with an intraclass correlation coefficient (ICC) 95% CI of 0.917–0.975 and an agreement estimated by Minimal Detectable Change (MDC_95_) between 8.6 and 10.8°. Interrater reliability was moderate to good, with an ICC 95% CI of 0.719–0.899 [26].

#### 1.3.2 Straight Leg Raise

For the SLR measurement, the participant also lay supine on the examination table and the EasyAngle® digital inclinometer was first calibrated to zero degrees on the surface of the table. The rater then explained the measurement procedure to the participant. The participant was asked to stay relaxed while the rater slowly raised their extended limb. The contralateral limb rested in extension and was stabilized by the rater's hand if necessary. The participant was asked to say “stop” at the moment of the first stretch sensation or pain onset and rater stopped the movement and recorded the angle. Like for the AKE measurement, the inclinometer was placed on the tibial crest, directly below the tibial tuberosity (Fig. 2, B).

The SLR is a passive HM flexibility measurement that is frequently performed with a universal goniometer or an analogue inclinometer. It has acceptable reliability and agreement [10,27]. Our reliability assessment in 90 people with CLBP found excellent intrarater reliability, with an ICC 95% CI of 0.908–0.987 and an MDC_95_ between 6.8 and 10.4°. The interrater ICC 95% CI was 0.775–0.907, indicating good reliability [26].

Fingertip-to-Floor distance.

The conventional procedure was used for the FTF measurement [28]. Participants stood with their feet together and bent forward with the knees extended, they were asked to reach as far as possible to the floor with their fingertips. The fingertip to floor distance was then measured by the rater using a standard measurement tape (Fig. 2, C).

This measurement is widely used to measure HM and spinal extensor muscle flexibility in people with CLBP, and it has good measurement proporties [10,28].

Participants completed the following questionnaires before performing the HM flexibility measurements: Oswestry Disability Index (ODI) [29], Fear Avoidance Belief Questionnaire (FABQ) [30], and Hospital Anxiety and Depression Scale (HADS) [31]. The maximum score for the ODI is 100 points, indicating complete disability. Maximum score for the FABQ physical activity subscore is 24 points and for the work subscore is 42 points. For the HADS, the maximum anxiety subscore is 21 and the maximum depression subscore is 21.

#### 1.3.3 Pain

Before and immediately after the intervention, participants were asked to rate the intensity of their low back pain on a Visual Analog Scale (VAS). Additionally, the presence of neuropathic pain was evaluated at baseline using the DN4 questionnaire [32]. A score of 4 points or more indicates a neuropathic component of the CLBP [33].

## 2 Intervention

### 2.1 Passive hamstring muscle stretching

Passive HM stretching was performed by a physical therapist experienced in the treatment of CLBP and trained in the study protocol. A simple, passive procedure was used to facilitate standardization and enhance repeatability. The procedure was based on stretching in manual therapy standards [22]. First, the physical therapist explained the procedure to the participant and reassured them that the stretching could be stopped if it was too hard to tolerate. With the participant lying supine, the stretched limb was placed in maximal, pain free hip flexion with the knee flexed, then the physical therapist slowly and gradually extended the knee to the maximal pain free position. In this position, participants felt an intense stretching sensation in the back of the thigh. If the position caused pain in the lower back, the hip flexion was reduced to the maximal, pain free ROM. A stopwatch was used to ensure the final position was held for 1 min for each limb.

## 3 Outcomes

The primary outcome was AKE angle. The mean value of the two measurements taken by Rater 2 before HM stretching were compared to the mean value of the two measurements taken by Rater 2 after the HM stretching. The secondary outcomes, SLR and FTF were compared in the same manner. The ODI, FABQ and HADS scores were collected at baseline and correlations with the change in AKE angle, SLR angle and FTF distance after HM stretching were analyzed.

### 3.1 Statistical analysis

For the primary outcome (change in AKE angle), a sample size calculation found that 90 participants were required to achieve effect-sizes above 0.2 with a two-sided type I error of 5%, statistical power above 80% and within-subject correlation of 0.8. This sample size was also appropriate to evaluate the secondary outcomes with satisfactory statistical power: (i) the correlations between psychosocial factors and change in HM flexibility and (ii) intra- and inter-rater reproducibility using ICCs.

Continuous data are presented as mean ± standard deviation. The normality of the distribution was assessed using the Shapiro-Wilk test. The paired Student t-test or Wilcoxon test was used to analyze change in pain intensity and change in flexibility measurements (AKE, SLR and FTF). The equality of variances was analyzed using Pitman's test. The relationships between continuous variables (ie, between change in AKE, SLR and FTF; and FABQ and HADS scores were analyzed with Pearson or Spearman's correlation coefficients, as appropriate. All statistical analyses were performed using version 15 Stata software, (StataCorp, College Station, TX, US). The tests were two-sided with a type I error set at 5%. Cohen's d was calculated to determine the effect sizes, which were interpreted as small (ES: 0.2), medium (ES: 0.5) and large (ES: 0.8, “grossly perceptible and therefore large”).

## 4 Results

**Fig. 3:**
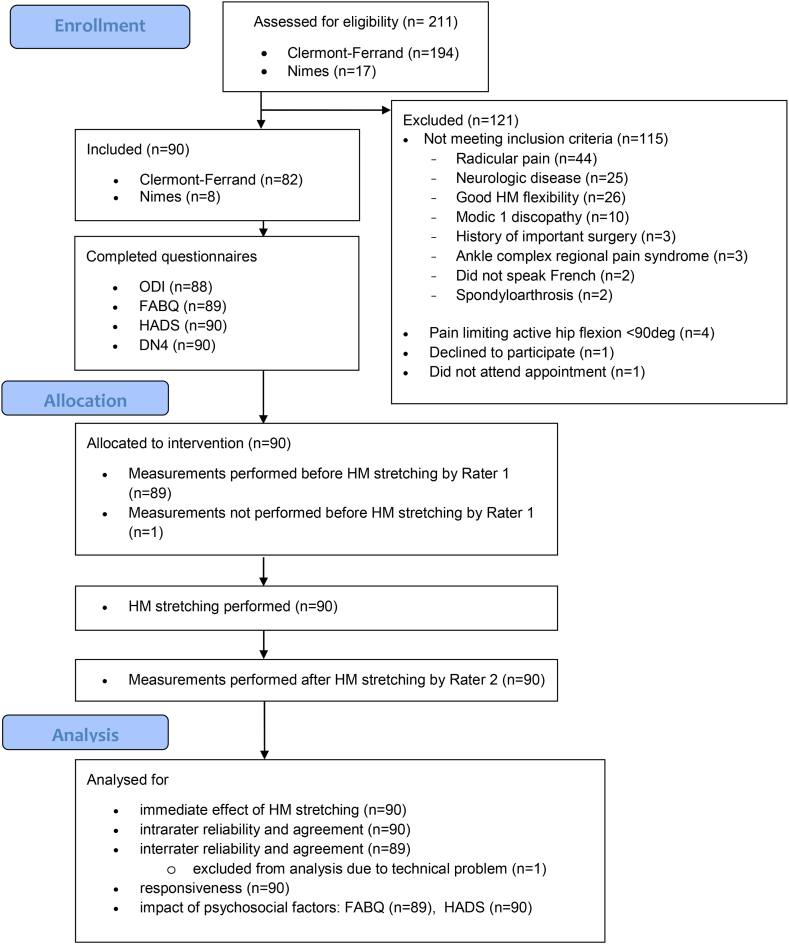
Flow diagram.

Participants were recruited from July 3rd^,^ 2020 to May 5th^,^ 2022. The overall study duration was 22 months. Ninety people with CLBP were included. Eight physical therapists participated either as raters or therapists for the application of the HM stretching. Their clinical experience in the management of CLBP ranged from 2 to 16 years. Five physical medicine and rehabilitation doctors participated in the recruitment procedure (including diagnosing the CLBP) and as raters; their experience in the management of CLBP ranged from 2 to 5 years. No serious adverse events occurred during the study. The recruitment process is presented in a flow diagram (Fig. 3), and participant characteristics are shown in Table 1. The number of participants included in the primary objective analysis was 90 (100%). For the secondary analysis, missing data were the consequence of non-response to the ODI (n = 2) and FABQ (n = 1).

**Table 1:** Sample characteristics.

Age [years]	44.4 ± 9.1
Men/women [n (%)]	52/38 (58/42)
Height [m]	171.2 ± 7.9
Mass [kg]	79.8 ± 18.3
BMI [kg/m^2^]	27.2 ± 5.7
Education level [n (%)]: Less than baccalaureate Baccalaureate level Higher education studies	45 (50) 32 (36) 13 (14)
Type of work [n (%)]: Sedentary Physical Mixed	22 (24) 48 (53) 20 (23)
	
Active smoking [n (%)]: Yes No	37 (41) 53 (59)
	
Workplace accident [n (%)]: Yes No	17 (19) 73 (81)
Time since pain onset [months]	81.0 ± 91.2
ODI FABQ PA FABQ W HAD A HAD D DN4	33.7 ± 12.7 14.0 ± 5.9 27.1 ± 11.4 9.3 ± 3.9 8.1 ± 3.3 4.4 ± 6.3
BMI, Body Mass Index; ODI, Ostwestry Disability Index; FABQ, Fear-Avoidence Beliefs Questionnaire; PA, physical activity; W, work; HAD, Hospital Anxiety and Depression scale; A, anxiety; D, depression; DN4, neuropathic pain questionnaire.	

Of the 211 people with CLBP screened, 26 (12%) had good HM flexibility, indicating a high prevalence of HM tightness among those in our physical medicine and rehabilitation departments. Mean ODI score was 33.7 ± 12.7, indicating moderate disability. Pain increased slightly after the intervention (mean VAS before 38 ± 2 versus mean VAS after 42 ± 2, p = 0.026*,* ES = 0.17). The scores of the FABQ and HADS are shown in Table 1.

**Fig. 4:**
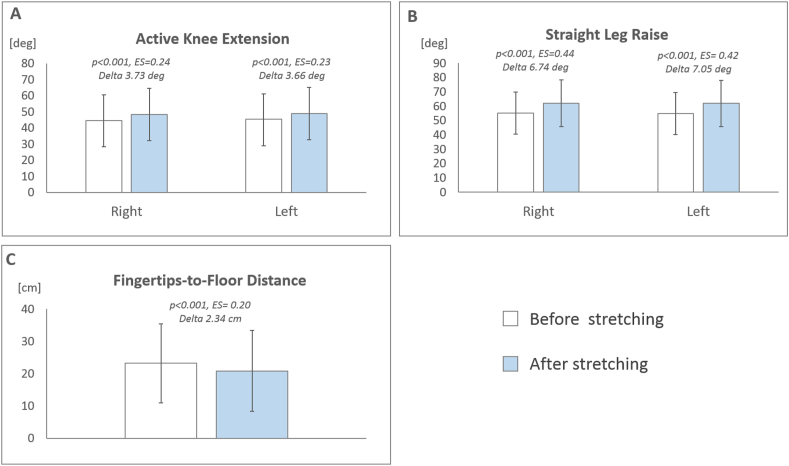
Immediate effect of hamstring muscle stretching on Active Knee Extension angle (A), Straight Leg Raise angle (B) and Fingertip-to-Floor distance (C) in 90 people with chronic low back pain.

All three HM flexibility measurements improved significantly after stretching. Mean improvement in AKE angle was 4°, 95% CI 2.4 to 5.1 (right AKE 44.7 ± 1.7 vs 48.4 ± 1.6, *p* < 0.001, ES = 0.24; left AKE 45.3 ± 1.7 vs 49.0 ± 1.6, p < 0.001, ES = 0.23) (Fig. 4, A), in SLR angle was 7°, 95% CI 5.5 to 8.6 (right SLR 55.2 ± 1.5 vs 62.0 ± 1.7, p < 0.001, ES = 0.44; left SLR 54.9 ± 16.3 vs 62.0 ± 17.4, p < 0.001, ES = 0.42) (Fig. 4, B), and in FTF distance was 2 cm, 95% CI 1.7 to 3.0 (FTF 23.2 ± 1.3 vs 20.9 ± 1.3, p < 0.001, ES = 0.20) (Fig. 4, C).

No significant correlations were found between change in any of the HM flexibility measurements and the FABQ or HADS scores (p > 0.05).

## 5 Discussion

This is the first study to determine the immediate effect of HM stretching on AKE and SLR, measured with a new digital inclinometer, and FTF in people with CLBP. All three outcomes improved significantly after 1 min of passive HM stretching, although the effect size was small. Contrary to our hypothesis, there was no relationship between the HM flexibility outcomes and psychosocial factors as measured by FABQ and HADS.

Our study confirms that HM stretching immediately improves HM flexibility. Nishikawa et al. found an immediate improvement in AKE angle of 15.8° after passive HM stretching, which is much greater than in our study [19]. However, their study was conducted in healthy individuals; in our study, CLBP and associated psychosocial factors could have limited the improvement in HM flexibility following stretching either by inhibiting motor control mechanisms, decreasing stretch tolerance [[34], [35], [36], [37]] or by fear and anxiety symptoms. Similarly, a greater immediate effect of HM stretching on FTF distance was found in healthy individuals than in our study (mean difference 5–6 cm) [18]. In people with non-specific LBP, the sit and reach test results improved immediately after HM stretching procedures [21]. This is in line with our results; however, the small sample size and different measurement technique limits the interpretation of their results. Interpretation of the results according to the minimal clinical important difference is necessary to define their clinical relevance, however no such values have been determined for the AKE, SLR or FTF. We estimated the MDC with 95% confidence bounds for the AKE and SLR [26]. The agreement for the FTF was previously determined [28]. According to these values, the changes in AKE angle and FTF distance may have resulted from measurement error. However, the effect size for the change in SLR angle was nearly twice that of AKE and FTF, and was at the limit of the MDC_95_ thus, the change is more likely to be of clinical importance. The SLR measurement was passive which may explain why it improved more after passive stretching than the active AKE and FTF measurements in which activation of the neuromuscular system is needed [38]. This suggests that the neuromuscular system needs active stimulation to generate active movement in the newly acquired passive ROM. This hypothesis is supported by the existence of both local and global neuromuscular control impairments, which are largely documented in CLBP [[34], [35], [36]].

We had hypothesized that protective behavior due to fear of pain, kinesiophobia or motivational aspects would limit changes in flexibility in some individuals. Evidence in the literature suggests that fear-avoidance beliefs are a prognostic factor for poor outcomes in people with LBP [39]. Furthermore, it has been reported that anxiety and depression limit the efficacy of a multidisciplinary intervention in people with CLBP [40]. However, in our study, the improvement in HM flexibility was not related to FABQ or HADS scores. This suggests that either psychosocial factors do not impact the immediate effect of HM stretching, or other approaches are required to evaluate these aspects. More direct human emotion recognition methods, using for example electroencephalography, galvanic skin response or heart rate variability, could yield different results [41]. This could be considered in further studies. This is the first study to evaluate a relationship between the presence of psychosocial factors and the immediate effect of HM stretching; no relationship was found.

### 5.1 Study limitations

This study has several limitations. First, to facilitate the feasibility of the study, we did not include a control group. However, the repeated measurements before and after HM stretching and the large sample size strengthen our results. Second, the measurement techniques do not directly measure change in HM flexibility, as ultrasound measurement would [42]. This and other measurements (eg, HM stiffness, HM electromyography or elastography) could be considered in further studies to better understand the effect of HM stretching. Also, only the FABQ and HADS were used to explore the impact of psychosocial factors. Other questionnaires or more direct measures of fear and stress could reveal different results thus could be used in future studies. Furthermore, in our study only immediate effect of HM stretching was evaluated, the impact of psychosocial factors could by different on cumulative effect of multiples stretching sessions.

## 6 Conclusion

Passive HM stretching immediately improved HM flexibility in people with CLBP. The AKE angle, SLR angle and FTF distance improved significantly; however only the change in passive SLR angle was clinically important, the changes in the active tests were not. In contrast with our hypothesis, psychosocial factors were not related to the magnitude of improvement in flexibility after HM stretching. Other methods of psychosocial evaluation could be considered in future studies. We recommend using passive HM stretching in addition to other interventions to obtain clinically important improvements in both passive and active movements.

## Funding

This research was funded by Centre Hospitalo-Universitaire (10.13039/501100015613CHU) de Clermont Ferrand and Université Clermont Auvergne but without a specific grant.

## Declaration of competing interest

The authors declare that they have no known competing financial interests or personal relationships that could have appeared to influence the work reported in this paper.
