# Supplementary material for: Immediate effect of passive hamstring stretching on flexibility and relationship with psychosocial factors in people with chronic low back pain
Source: Heliyon. 2023 Sep 4;9(9):e19753. doi: 10.1016/j.heliyon.2023.e19753 (PMC10559058; doi:10.1016/j.heliyon.2023.e19753)
Supplement: Multimedia component 1 [file mmc1.docx]

**Article Publishing Charge**

Yes, I have read and understood the information regarding open access and understand that I have to pay an article publication fee upon acceptance of my manuscript.

**Category**

**Medical Science**

Clinical Research

**Publication ethics**

Please confirm that you have reviewed our guidelines for [Ethics in Publishing](https://www.elsevier.com/about/policies/publishing-ethics).

I confirm

**Clinical Study**

Does your study include a clinical study? Yes

Is your study an (interventional) clinical trial or an observational study? Clinical Trail

Please provide name of registry and clinical trial registration number.

ClinicalTrials.gov database, registration number: NCT04551326

Please confirm that experiments have been conducted according to the [CONSORT](http://www.consort-statement.org/) guidelines. Yes

**Animal Experiments**

Does your research involve experimentation on animals? No

**Human Subjects**

Does your study include human subjects? Yes

Please provide the name of the ethical committee approving these experiments.

Comité de Protection des Personnes – Ouest 1, Identifier: 2020T2-01_RIPH2 HPS_2019-A03000-57

Please confirm authors’ compliance with all relevant ethical regulations. Yes

Please confirm that written consent has been obtained from all patients/participants. Yes

**Data Availability**

Has data associated with your study been deposited into a publicly available repository? No

Please select why. Please note that this statement will be available alongside your article upon publication.

Data will be made available on request.

**Competing Financial Interests**

We, the authors and our immediate family members, have no financial interests to declare.

**Advisory/Management and Consulting Positions**

We, the authors and our immediate family members, have no positions to declare and are not members of the journal’s editorial teams or advisory boards.

**Patents**

We, the authors and our immediate family members, have no related patents to declare.

Please insert any “Declaration of Interests” statement in this space.

The authors declare no competing interests.

**Heliyon Null Hypothesis**

No, my manuscript does not contain “negative” or “inconclusive” results.

**Special Issue**

Is your manuscript being submitted to a special issue? No

**Double anonymized peer review**

When your paper is sent for external peer review, should peer reviewers be able to see you and your co-authors' identities?

**No, I request double anonymized peer review for my manuscript. I have removed all identifying details from all submission files and only included my authorship list in in the cover letter. If you have not yet prepared your manuscript and auxiliary files in this way, please return to the Attach Files stage and replace the versions uploaded so far with updated ones.**

**Institutional Affiliations**

All affiliations for all co-authors are listed on the title page of the manuscript.

Do you have any related research objects (data, methods, protocols, software, hardware) that you would like to publish alongside your original research as an additional paper in [Research Elements](https://www.elsevier.com/authors/tools-and-resources/research-elements-journals)? No
